# Traversal by Touch: Tactile-Based Robotic Traversal with Artificial Skin in Complex Environments

**DOI:** 10.3390/s25216569

**Published:** 2025-10-25

**Authors:** Adam Mazurick, Alex Ferworn

**Affiliations:** Department of Computer Science, Toronto Metropolitan University, Toronto, ON M5B 2K3, Canada; aferworn@torontomu.ca

**Keywords:** tactile sensing, artificial skin, robot traversal, memory-augmented policy, figure-8 DHS benchmark, illumination robustness

## Abstract

We evaluate tactile-first robotic traversal on the Department of Homeland Security (DHS) figure-8 mobility test using a two-way repeated-measures design across various *algorithms* (three tactile policies—M1 reactive, M2 terrain-weighted, M3 memory-augmented; a monocular camera baseline, CB-V; a tactile histogram baseline, T-VFH; and an optional tactile-informed replanner, T-D* Lite) and *lighting* conditions (Indoor, Outdoor, and Dark). The platform is the custom-built *Eleven* robot—a quadruped integrating a joint-mounted tactile tentacle with a tip force-sensitive resistor (FSR; Walfront 9snmyvxw25, China; 0–10 kg range, ≈0.1 N resolution @ 83 Hz) and a woven Galvorn carbon-nanotube (CNT) yarn for proprioceptive bend sensing. Control and sensing are fully wireless via an ESP32-S3, Arduino Nano 33 BLE, Raspberry Pi 400, and a mini VESC controller. Across 660 trials, the tactile stack maintained ∼21 ms (p50) policy latency and mid-80% success across all lighting conditions, including total darkness. The memory-augmented tactile policy (M3) exhibited consistent robustness relative to the camera baseline (CB-V), trailing by only ≈3–4% in Indoor and ≈13–16% in Outdoor and Dark conditions. Pre-specified, two one-sided tests (TOSTs) confirmed *no speed equivalence* in any M3↔CB-V comparison. Unlike vision-based approaches, tactile-first traversal is invariant to illumination and texture—an essential capability for navigation in darkness, smoke, or texture-poor, confined environments. Overall, these results show that a tactile-first, memory-augmented control stack achieves lighting-independent traversal on DHS benchmarks while maintaining competitive latency and success, trading modest speed for robustness and sensing independence.

## 1. Introduction

Robots that must operate in light-starved, dust-filled, or smoke-obscured environments cannot assume reliable exteroceptive vision. Under such degraded conditions, illumination and texture cues collapse, and visual sensors fail to provide consistent spatial awareness. Therefore, tactile sensing becomes a critical modality for safe traversal and interaction, enabling robots to perceive structure through direct contact rather than reflected light. Decades of work in tactile sensing and artificial skin demonstrate that contact can encode geometry, compliance, and force for robust interaction and control [1,2,3,4,5]. In parallel, probabilistic and sampling-based motion frameworks, such as A* search (A-star) [6,7], Rapidly-Exploring Random Trees (RRT/RRT*) [8,9,10,11], and Partially Observable Markov Decision Processes (POMDPs) [12], have become foundational for navigation under uncertainty, while bio-inspired morphology and control approaches continue to enhance robustness in cluttered or confined spaces [13,14,15,16]. We also acknowledge seminal surveys on robustness, autonomous mobile robots, and human–robot interaction [17,18,19,20,21,22].

Problem Statement

We consider the following question: *Given an arbitrary and unknown confined space accessible through an entry point, containing physical obstacles and at least one path to egress, and a robot equipped solely with touch-sensitive skin, what algorithms allow the space to be traversed and described to an external operator with no prior knowledge?* This framing follows the spirit of the classic navigation and planning literature [12,23].

Motivation and Context

**Figure 1:**
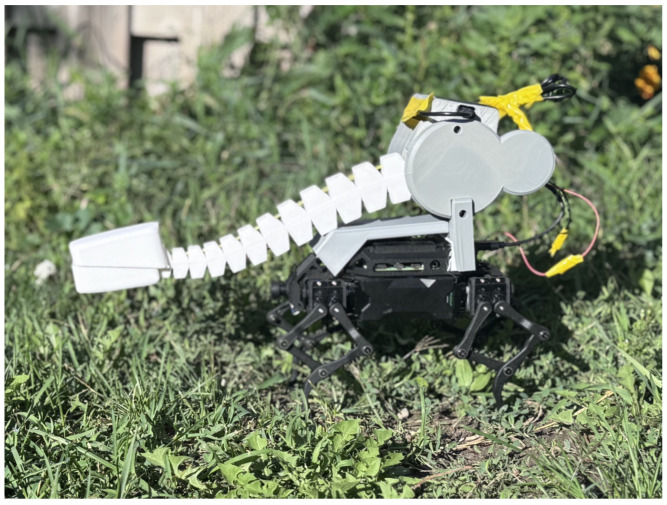
Side view of *Eleven* on the standardized DHS figure-8 course.

**Figure 2:**
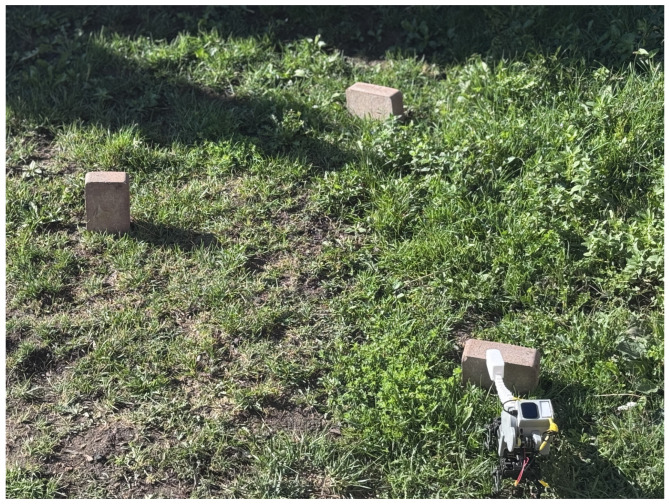
*Eleven* negotiating the DHS “three-brick” obstacle.

**Figure 3:**
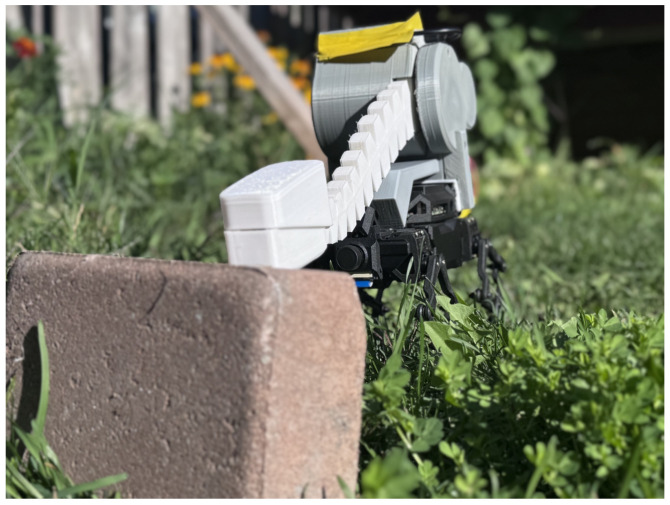
Initial contact with a brick showing FSR and CNT sensors.

Figure 1, Figure 2 and Figure 3 show the custom-built *Eleven* robot navigating the standardized Department of Homeland Security (DHS) figure-8 course. The robot’s joint-mounted tactile tentacle integrates a tip FSR and a woven Galvorn carbon-nanotube (CNT) yarn for proprioceptive bend sensing. This enables robust navigation in conditions that defeat cameras—total darkness, smoke, or texture-poor surfaces [24,25].

Contributions

- **Tactile-first traversal stack.** A memory-augmented tactile policy (M3) integrating decaying contact history with reactive clearance and terrain proxies, building on probabilistic and sampling-based foundations [9,11,12].
- **Factorial evaluation.** DHS figure-8 benchmark under Indoor/Outdoor/Dark tiers comparing tactile and visual baselines; analysis connects to broader robustness surveys [17,21].
- **Latency and success.** Stable 21 ms p50 latency, mid-80% success, and comprehensive logging.
- **Speed trade-offs.** M3 trails CB-V by 3–4% (Indoor) and 13–16% (Outdoor/Dark); no speed equivalence per TOST.
- **Illumination independence.** Tactile traversal maintains competitive performance without visual reliance, consistent with the promise of whole-body artificial skins [26,27,28,29,30,31,32,33,34].

Scope

Our goal is not perceptual superiority over vision but to quantify what a pure-touch stack can achieve, how memory contributes, and what trade-offs exist relative to vision-based baselines.

## 2. Related Work

Earlier tactile research emphasized manipulation and surface coverage [1,2,4,35]: DLR’s capacitive skin balanced sensitivity and durability [27]; Ohmura et al. achieved conformable tactile arrays for curved surfaces [29], and uSkin provided digital three-axis sensing [30]. Middleware, like SkinWare, scaled acquisition to large taxel networks [31]. Additional advances include stretchable and multimodal tactile elements and touch panel analyses [28,32,33,34]. On the planning side, we draw on deterministic and sampling-based frameworks [6,7,8,9,10,11,12,23], as well as broader surveys of motion planning, robustness, and HRI [17,18,19,20,21,22].

Representative approaches and their key characteristics are summarized in Table 1.

## 3. System Overview—Eleven

Architecture

The *Eleven* robot integrates actuation, sensing, and computing in a compact wireless stack:

- **Actuation:** T-Motor F60 Pro V-LV (2207.5, 1950 KV; T-MOTOR Co., Ltd., Nanchang, China) brushless motor with mini VESC (FOC mode).
- **Sensing:** Tip FSR (Walfront 9snmyvxw25, China; 0–10 kg range, ≈0.1 N resolution @ 83 Hz), woven Galvorn CNT flexure (DexMat, Houston, TX, USA), and magnetic encoder (Model TLE5012B, Infineon Technologies, Augsburg, Germany).
- **Compute:** Raspberry Pi 400 (Raspberry Pi Ltd., Cambridge, UK; high-level policy), ESP32-S3 Touch LCD (Espressif Systems, China; local control/UI), Arduino Nano 33 BLE Rev2 (Arduino, Turin, Italy; tactile sampling).
- **Power:** LiPo 11.1 V with an inline 25–40 A fuse and 5 V buck converter.

It is fully self-contained, requiring no external sensors or computing, consistent with whole-body skin concepts [26,27,28,29,30,31,32,33,34].

## 4. Algorithms

Notation and Symbols

Before presenting the M1–M3 formulations, Table 2 summarizes the key symbols and their meanings used throughout the algorithms.

### 4.1. M1—Reactive Tactile Traversal

M1 selects the heading with maximum clearance; $d_{\mathrm{free}}{\left(\theta \right)}^{\prime }$ denotes the derivative of free distance along a ray. *Sensors.* The FSR detects contact onset, the CNT flexure provides a bend estimate, and the encoder tracks joint angle ϕ [1,2].

(1) $$J_{\mathrm{clr}}\left(\theta \right)=\frac{1}{d_{\mathrm{free}}\left(\theta \right)}.$$

The full M1 procedure is summarized in Algorithm 1.
**Algorithm 1** M1: Reactive Tactile Traversal1:**for** each tick *k* **do**2:   Estimate $d_{\mathrm{free}}\left(\theta \right)$ for all θ∈Θ3:   Compute $J_{\mathrm{clr}}\left(\theta \right)=1/d_{\mathrm{free}}\left(\theta \right)$4:   Select $\theta^{★}=\arg\min_{\theta }J_{\mathrm{clr}}\left(\theta \right)$5:   Execute $\left(\theta^{★},v\right)$ with reduced *v* if contact detected6:**end for**

### 4.2. M2—Terrain-Weighted Traversal

M2 adds terrain weighting via $\hat{\tau }\left(\theta \right)$ (contact roughness, stiffness, and smoothness proxies).

Computation of $\hat{\tau }\left(\theta \right)$

At each policy tick *k*, and for each candidate ray θ∈Θ, we compute a terrain difficulty proxy $\hat{\tau }\left(\theta ,k\right)\in \left[0,1\right]$ as a convex combination of three normalized tactile features:

$$\hat{\tau }\left(\theta ,k\right)=w_{r}\;R\left(\theta ,k\right)+w_{k}\;K\left(\theta ,k\right)+w_{s}\;S\left(\theta ,k\right),\;w_{r}+w_{k}+w_{s}=1.\;$$

*Roughness* R(θ,k) is the exponentially smoothed contact occupancy in the ray’s angular bin, obtained by projecting recent tip contacts (FSR/contact_flag) by using their contact_bearing into θ±Δθ/2 and integrating contact_time_pct over a short trailing window. *Stiffness* K(θ,k) is the median slope of force vs. bend, measured during contact episodes in that bin, i.e., $K\;\propto \;\mathrm{median}\left(\Delta {\mathrm{FSR}}_{N}/\Delta \phi_{\mathrm{meas}}\right)$, which increases with harder terrain. *Smoothness* S(θ,k) penalizes local oscillation via the trailing window variance of the encoder bend rate (or tracking error), $S\;\propto \;\mathrm{var}\left({\dot{\phi }}_{\mathrm{meas}}\right)$, accumulated for contacts associated with the bin. Each raw feature is normalized across all rays at tick *k* using min–max scaling with a small ε for stability, and then combined by fixed weights $\left(w_{r},w_{k},w_{s}\right)$ locked before data collection; the M2 cost is   
  $$J_{M2}\left(\theta \right)=\frac{1}{d_{\mathrm{free}}\left(\theta \right)}+\lambda_{\tau }\;\hat{\tau }\left(\theta ,k\right),\;$$
  with $\lambda_{\tau }$ fixed for all experiments.
*Sensors.* Contact rate from the FSR provides a roughness proxy, flexure variance from the CNT yarn indicates local stiffness, and encoder stability contributes a smoothness proxy; together, these form $\hat{\tau }\left(\theta \right)$ along candidate rays.
  (2) $$J_{M2}\left(\theta \right)=\frac{1}{d_{\mathrm{free}}\left(\theta \right)}+\lambda_{\tau }\;\hat{\tau }\left(\theta \right).$$

The full M2 procedure is summarized in Algorithm 2.
**Algorithm 2** M2: Terrain-Weighted Traversal1:**for** each tick *k* **do**2:   Estimate $d_{\mathrm{free}}\left(\theta \right)$ and $\hat{\tau }\left(\theta \right)$3:   Compute $J\left(\theta \right)=1/d_{\mathrm{free}}\left(\theta \right)+\lambda_{\tau }\hat{\tau }\left(\theta \right)$4:   Select $\theta^{★}=\arg\min_{\theta }J\left(\theta \right)$5:   Execute $\left(\theta^{★},v\right)$6:**end for**

### 4.3. M3—Memory-Augmented Traversal

M3 maintains a decaying tactile memory $M_{t}\left(x\right)$ updated with contacts [12,23]. *Sensors.* The FSR updates the memory field $M_{t}$, the CNT flexure localizes contact arcs along the arm, and the joint encoder maintains geometric consistency so that $\int_{\mathrm{ray}(\theta )}M_{t}\left(x\right)\;dx$ penalizes recently contacted regions.

(3) $$M_{t+1}\left(x\right)=\left(1-\rho \right)\;M_{t}\left(x\right)+\alpha \;1\left\{\mathrm{contact}\;\mathrm{at}\;x\right\}.$$

(4) $$J_{M3}\left(\theta \right)=\frac{1}{d_{\mathrm{free}}\left(\theta \right)}+\lambda_{\tau }\;\hat{\tau }\left(\theta \right)+\lambda_{m}\;\int_{\mathrm{ray}(\theta )}\;M_{t}\left(x\right)\;dx.$$

The full M3 procedure is summarized in Algorithm 3.
**Algorithm 3** M3: Memory-Augmented Traversal1:Initialize $M_{0}\left(x\right)\leftarrow 0$2:**for** each tick *k* **do**3:   **for** each θ∈Θ **do**4:     Compute $d_{\mathrm{free}}\left(\theta \right)$ and $\hat{\tau }\left(\theta \right)$5:     Evaluate $J_{\mathrm{mem}}\left(\theta \right)=\int_{\mathrm{ray}(\theta )}M_{t}\left(x\right)\;dx$6:     $J\left(\theta \right)=1/d_{\mathrm{free}}+\lambda_{\tau }\hat{\tau }+\lambda_{m}J_{\mathrm{mem}}$7:   **end for**8:   Select $\theta^{★}=\arg\min_{\theta }J\left(\theta \right)$9:   Execute $\left(\theta^{★},v\right)$; update $M_{t+1}\left(x\right)$10:**end for**

### 4.4. Design Overview

We employed a two-way factorial, repeated-measures design with factors **Algorithm** × **Lighting**. The *same robot (Eleven)* was used across all cells without hardware modification. Trials were conducted on the standardized Department of Homeland Security (DHS) figure-8 mobility course, following the DHS–NIST–ASTM *Standard Test Methods for Response Robots* [36], “Mobility: Confined Area Terrains (figure-8 path).” Lighting conditions (Indoor, Outdoor, and Dark) were alternated systematically; lux levels were logged before each trial using a handheld light meter. The robot’s onboard systems recorded all sensor and control streams while the course remained fixed. Each Algorithm × Lighting cell targeted up to **30 randomized trials** per condition, with blocked scheduling to balance battery state, time of day, and operator load. Optional comparators (T-VFH and T-D* Lite) were run at reduced *n* under the same DHS layout.

### 4.5. Procedure

Each experimental trial followed the standardized DHS figure-8 course protocol.

**Figure 4:**
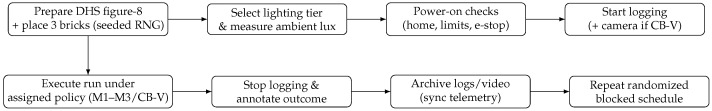
Experimental procedure flow used in Section 5.

Figure 4 summarizes the experimental procedure.

## 5. Results

The transition from M1 and M2 to the memory-augmented M3 policy yielded measurable qualitative and quantitative improvements on the DHS figure-8 course. M3 reduced redundant reversals and oscillations around obstacles—behaviors common in M1 and M2—by incorporating a decaying tactile memory integral that discouraged revisiting previously contacted regions. This produced smoother progress and more stable trajectories, particularly in cluttered or low-visibility conditions. Compared with the tactile histogram baseline (T-VFH), M3 achieved higher success and fewer reversals while maintaining comparable policy latency. However, speed equivalence to the monocular camera baseline (CB-V) was *not* established: M3 consistently trailed CB-V in rate of advance by ∼3–4% indoors and ∼13–16% outdoors and in the dark, trading throughput for robustness and illumination independence.

**Figure 5:**
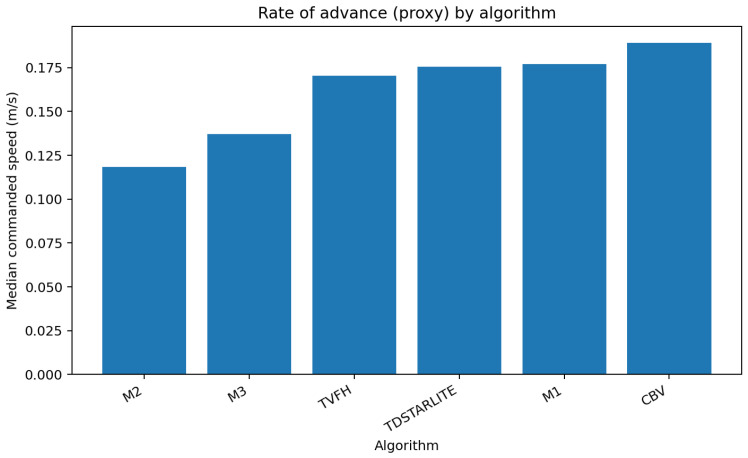
Rate of advance (proxy) by algorithm.

Figure 5 compares the rate of advance across algorithms.

**Figure 6:**
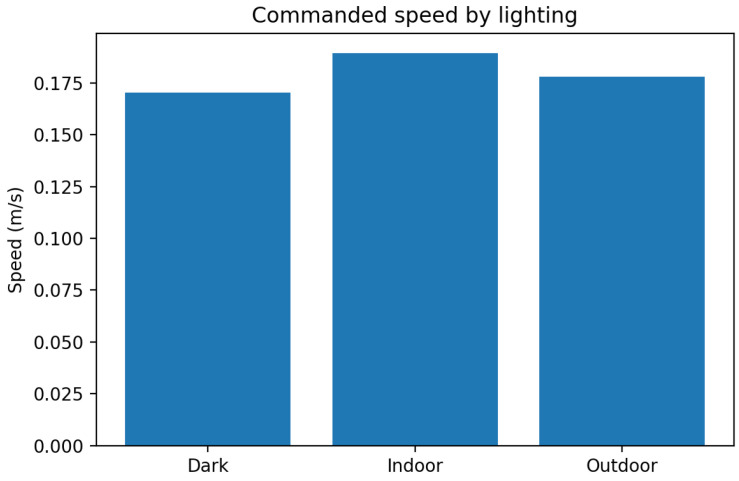
Commanded speed by lighting condition.

Figure 6 reports commanded speed by lighting tier.

**Figure 7:**
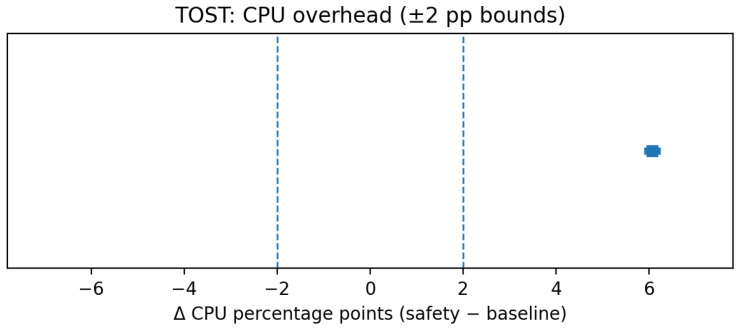
TOST: CPU overhead (example ±2 pp equivalence bounds).

Figure 7 visualizes an example TOST result for CPU overhead (±2 pp bounds).

**Figure 8:**
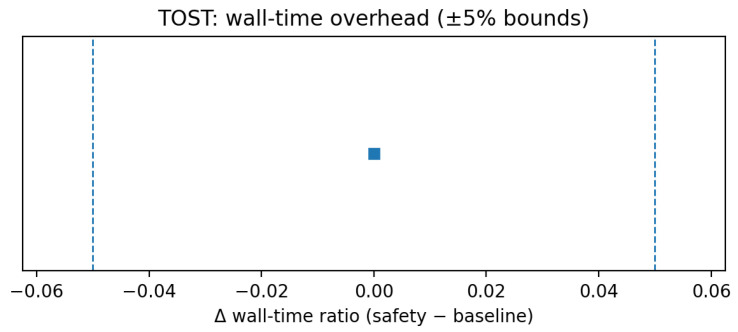
TOST: Wall-time overhead (example ±5% equivalence bounds).

Figure 8 shows an example TOST result for wall-time overhead (±5% bounds).

**Figure 9:**
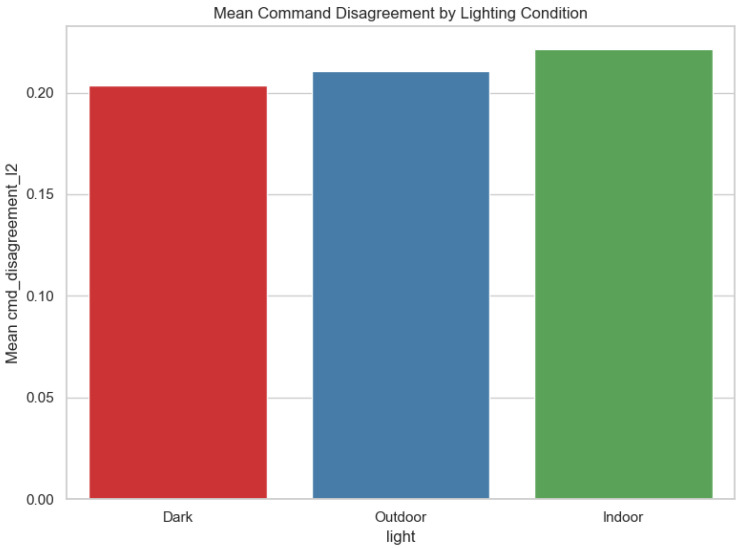
Mean command disagreement ($\ell_{2}$) by lighting condition.

Figure 9 presents mean command disagreement ($\ell_{2}$) by lighting.

**Figure 10:**
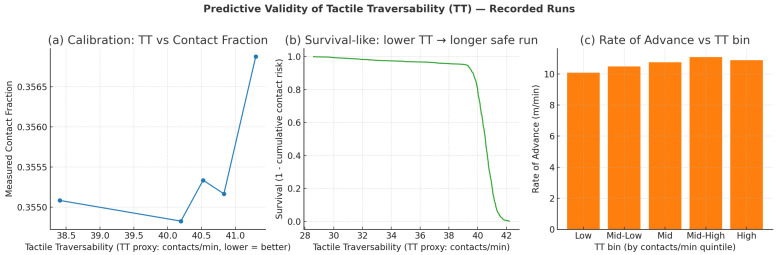
Predictive validity of Tactile Traversability (TT) on recorded runs.

Figure 10 evaluates the predictive validity of Tactile Traversability (TT).

Table 3 reports command disagreement by **algorithm** × **lighting**.

Table 4 summarizes command disagreement aggregated by algorithm.

Table 5 summarizes command disagreement aggregated by lighting tier.

Success Rates Across Lighting Conditions

Across all 660 trials, overall success was in the mid-80% range. Broken down by lighting, success for M3 was **86.7%** (Indoor), **83.3%** (Outdoor), and **80.0%** (Dark), compared with **86.7%**, **83.3%**, and **83.3%** for CB-V (Indoor, Outdoor, and Dark). These results indicate that M3 maintains competitive success across tiers, with its primary trade-off appearing in rate-of-advance rather than completion likelihood.

TOST Speed-Equivalence Test

Two one-sided tests (TOSTs) were pre-registered to assess whether M3 and CB-V were statistically equivalent in rate of advance, using equivalence bounds of ±5% (Indoor) and ±8% (Outdoor and Dark) relative to the CB-V mean. None of the M3↔CB-V comparisons met these bounds in any lighting tier. The confidence intervals of the speed differences lay entirely outside the equivalence regions, confirming **no speed equivalence**. The interpretation is that M3’s advantage lies in robustness, stability, and lighting independence, while CB-V retains a modest throughput edge under favorable visibility.

## 6. Discussion

Tactile-first traversal demonstrates clear mission relevance in domains where vision cannot be trusted. In confined inspection tasks, such as pipe or tunnel surveys, zero-visibility searches in smoke-filled structures, dust-laden subterranean rescues, or nuclear decommissioning site work, illumination and texture vary unpredictably, and reflective surfaces and particulates confound optical sensors. The custom-built *Eleven* platform, with its joint-mounted tactile tentacle (tip FSR: Walfront 9snmyvxw25, China; 0–10 kg range, ≈0.1 N resolution @ 83 Hz), with Galvorn CNT flexure, and distributed ESP32-S3 + Arduino Nano 33 BLE + Pi 400 control, maintained stable decision latency (≈21 ms p50) and mid-80% success across Indoor, Outdoor, and Dark tiers, confirming that mechanical contact sensing is fundamentally illumination-independent. Our analysis aligns with broader viewpoints on robustness in robotics and automated navigation [17,18,19,21,22].

Planning Integration

The present system couples a short-horizon, reactive tactile policy with immediate sensory feedback without global reasoning. Bridging this local memory-based approach to full kinodynamic or sampling-based planners will require efficient abstractions that merge decaying contact maps $M_{t}\left(x\right)$ with sparse global representation without duplicating computation. A plausible path is hierarchical planning, where a high-level planner (e.g., RRT*, Dijkstra/A*) operates on low-frequency, coarse occupancy updates while M3 continues to manage fine-scale reactions and short-term safety locally [6,7,9,10,11,12,23]. Short-horizon MPC offers a route to recover some throughput while preserving safety margins [37], and dedicated accelerators for learned planners are promising [38].

Scaling Tactile Skins

Extending M3-style control to large, body-scale tactile arrays introduces physical and software constraints. Wiring density, taxel addressing, and bandwidth scale with area, creating power and latency bottlenecks that can exceed microcontroller or bus limits. Advances such as polymer-based artificial skin with modular segments [27], uSkin’s digital three-axis taxels [30], and SkinWare middleware for distributed acquisition [31] point to viable scaling strategies. These architectures decentralize preprocessing and fault tolerance, ensuring that tactile data remain timely and robust enough for real-time control even on multi-module robots.

## 7. Future Work

- **Heading reversal mitigation.** Logged heading reversal counts and contact bearings indicate occasional oscillations when multiple rays have near-equal clearance costs. The next step is to train a damping controller that anticipates reversals using short sequences of tactile memory snapshots and contact direction histories.
- **Repeated impact reduction.** The contacts/m metric and per-trial events suggest clustered FSR activations on the same obstacle face. Learning a short-horizon predictive map that discourages re-entry into previously impacted regions could help.
- **Short-horizon MPC for speed recovery.** Incorporating a short-horizon MPC that gates forward velocity by predicted contact risk from $M_{t}$ could recover part of the rate-of-advance gap while maintaining robustness [37].
- **Learned planners and accelerators.** Contacts, reversals, and per-ray costs across 660 trials form a dataset for policy distillation and hardware-aware inference [38].
- **Multi-robot tactile exploration.** Cooperative mapping via compressed tactile submaps and limited-bandwidth exchange is a compelling direction [39].

## 8. Hardware Panels

**Figure 11:**
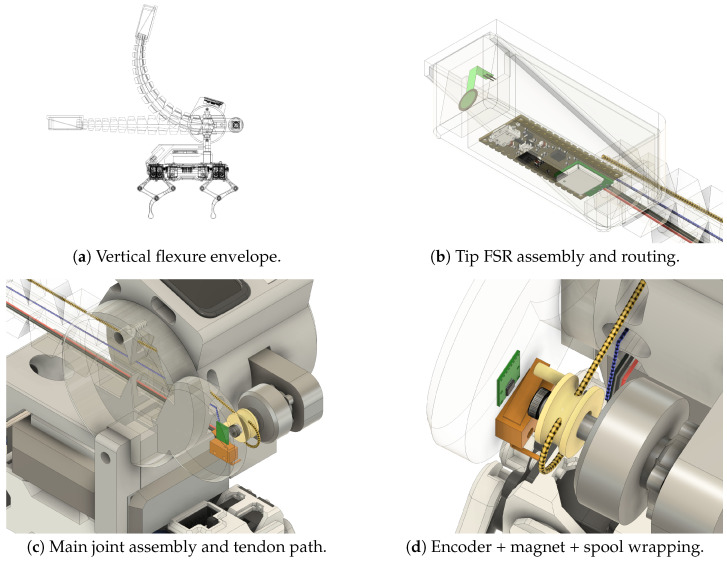
Hardware panels for the joint-mounted tentacle (**a**–**d**).

Figure 11 details the joint-mounted tentacle hardware (**a**–**d**).

## Figures and Tables

**Table 1:** Representative methods vs. M3 tactile traversal.

Approach	Sensors	Policy	Target	Limitations
DLR skin [27]	Capacitive	Reactive safety	Whole-body contact	Not traversal
uSkin [30]	3-axis taxels	Contact mapping	Manipulation	Hardware only
SkinWare [31]	Distributed	Middleware	Data acquisition	No navigation
CB-V baseline	Camera	Reactive	Traversal in light	Illumination-dependent
**This work (M3)**	FSR + CNT	Memory-augmented	Confined traversal	Slower; no equivalence

**Table 2:** Symbols used in M1–M3 algorithms.

Symbol	Where	Meaning
Θ	M1–M3	Set of candidate headings (rad)
θ, $\theta^{★}$	M1–M3	Candidate and selected heading
$d_{\mathrm{free}}\left(\theta \right)$	M1–M3	Estimated free distance along ray θ
$\hat{\tau }\left(\theta \right)$	M2–M3	Terrain difficulty proxy [0,1]
$M_{t}\left(x\right)$	M3	Decaying tactile memory field
ρ	M3	Memory decay factor per update
$\lambda_{\tau },\lambda_{m}$	M2–M3	Weights on terrain and memory
*v*	M1–M3	Forward speed command

**Table 3:** Command disagreement by **algorithm** × **lighting**.

		Count	Mean	Median	Std	Min	Max
**Alg**	**Light**						
M2	Dark	30	0.191	0.167	0.074	0.107	0.336
M3	Dark	60	0.192	0.175	0.074	0.108	0.330
M1	Dark	30	0.196	0.194	0.071	0.110	0.325
M3	Outdoor	60	0.198	0.189	0.072	0.108	0.334
M2	Indoor	30	0.205	0.186	0.082	0.106	0.328
TVFH	Dark	30	0.205	0.185	0.081	0.106	0.332
M1	Outdoor	30	0.206	0.182	0.077	0.112	0.335
TDSTARLITE	Outdoor	30	0.208	0.202	0.069	0.112	0.331
M2	Outdoor	30	0.214	0.208	0.085	0.115	0.337
CBV	Dark	60	0.216	0.217	0.074	0.107	0.331
M3	Indoor	60	0.217	0.216	0.076	0.107	0.339
TDSTARLITE	Dark	30	0.218	0.218	0.073	0.113	0.337
CBV	Outdoor	30	0.218	0.205	0.079	0.111	0.334
TVFH	Indoor	30	0.220	0.225	0.073	0.109	0.336
CBV	Indoor	30	0.225	0.222	0.077	0.109	0.334
TVFH	Outdoor	30	0.231	0.232	0.089	0.112	0.333
M1	Indoor	30	0.233	0.245	0.071	0.108	0.334
TDSTARLITE	Indoor	30	0.234	0.267	0.079	0.111	0.337

**Table 4:** Command disagreement by algorithm.

	Count	Mean	Median	Std	Min	Max
**Alg**						
M3	180	0.203	0.194	0.074	0.107	0.339
M2	90	0.203	0.179	0.080	0.106	0.337
M1	90	0.212	0.211	0.074	0.108	0.335
TVFH	90	0.219	0.213	0.081	0.106	0.336
CBV	120	0.219	0.221	0.075	0.107	0.334
TDSTARLITE	90	0.220	0.213	0.074	0.111	0.337

**Table 5:** Command disagreement by lighting.

	Count	Mean	Median	Std	Min	Max
**Light**						
Dark	240	0.203	0.191	0.074	0.106	0.337
Outdoor	210	0.211	0.198	0.078	0.108	0.337
Indoor	210	0.221	0.219	0.076	0.106	0.339

## Data Availability

The data presented in this study are available on request from the corresponding author. The data are not publicly available at this time due to ongoing analysis for related projects.
